# How to Control for Gestational Age in Studies Involving Environmental Effects on Fetal Growth

**DOI:** 10.1289/ehp.11105

**Published:** 2008-07

**Authors:** Rémy Slama, Babak Khoshnood, Monique Kaminski

**Affiliations:** Avenir Team “Environmental Epidemiology Applied to Fecundity and Reproduction”, INSERM U823, Grenoble, France, E-mail: remy.slama@ujf-grenoble.fr; Epidemiological Research Unit on Perinatal and Women’s Health, INSERM UMR S149, IFR 69, Villejuif, France

In studies on the effects of environmental factors on fetal growth, birth weight is usually corrected for gestational age. With the generalized use of ultrasound examinations in many countries, gestational age is often defined or corrected from the ultrasound measurements performed during or immediately after the first trimester of pregnancy, which are compared to a reference growth curve. As an illustration, in a cohort study investigating the association between exposure to perfluorinated chemicals and fetal growth, Fei et al. (2007) defined gestational age from ultrasound measures performed before 24 gestational weeks and, if this information was missing, from the date of the last menstrual period (LMP).

The superiority of ultrasound measurements over other approaches to predict the date of delivery (Lynch and Zhang 2007) does not imply that ultrasound-based gestational age leads to an unbiased estimate of the effect of environmental factors on fetal growth. The use of ultrasound-based gestational age assumes that fetal ultrasound measurements at a given gestational week during the first trimester have very little variability. However, there is some evidence to the contrary (Bukowski et al. 2007). Part of this variability might be due to exposure to environmental pollutants. If the environmental pollutant considered can restrict fetal growth as early as the first trimester, correcting gestational age using first-trimester ultrasound measurements will erroneously shorten the gestational age of these small-for-gestational-age fetuses. This may lead to underestimating effects of environmental pollutants on birth weight or size controlled for gestational age (Figure 1), compared with studies using an accurately estimated date of conception. In practice, an accurate estimate of conception date may seldom be available outside the setting of *in vitro* fertilization. An alternative is reliance on LMP-based estimates, which are prone to errors due to bad recall, variability in the duration of the follicular phase of the cycle and midcycle, and early pregnancy bleeding (Lynch and Zhang 2007). Moreover, using the LMP-based estimate of gestational age would be problematic if, as already reported for specific environmental pollutants (Windham et al. 2003), the environmental factors considered could influence the duration of the menstrual cycle. Therefore, detailed studies may be needed to determine the balance between the possible biases in the estimated effect of the environmental factor entailed by the use of ultrasound-based measurements and LMP-based estimates.

This potential bias has been recognized by Savitz et al. (2002) and was alluded to by Fei et al. (2007) in their “Discussion.” However, its consequences have probably not been fully acknowledged. When possible, researchers should conduct sensitivity analyses using different measures of gestational age to help quantify the potential for bias. The same approach could also be used when gestational duration is the studied outcome (Lynch and Zhang 2007).

## Figures and Tables

**Figure 1:**
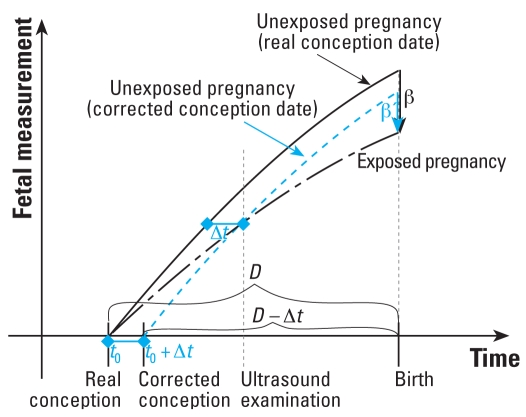
Hypothetical evolution of a fetal measurement (e.g., fetal length) during pregnancy for a pregnancy exposed or unexposed to an environmental factor that can affect fetal growth from early pregnancy. The ultrasound examination leads the obstetrician to correct the date of conception (*t*_0_) for the exposed pregnancy by Δ*t*, so this exposed pregnancy is not compared with unexposed pregnancies with the same gestational age D (solid curve) as it should, but instead with gestational age D – D*t* (dashed blue curve). Consequently, the estimated difference in the gestational age–specific fetal measurement at birth between exposed and unexposed pregnancies is not the correct value β but a smaller value β′.
